# Neuroprotective Effect of Xueshuantong for Injection (Lyophilized) in Transient and Permanent Rat Cerebral Ischemia Model

**DOI:** 10.1155/2015/134685

**Published:** 2015-11-23

**Authors:** Xumei Wang, Shaoxia Wang, Jinxin Wang, Hong Guo, Zhaopeng Dong, Lijuan Chai, Limin Hu, Yue Zhang, Hong Wang, Lu Chen

**Affiliations:** ^1^Tianjin State Key Laboratory of Modern Chinese Medicine, Tianjin University of Traditional Chinese Medicine, Tianjin 300193, China; ^2^Tianjin Key Laboratory of Chinese Medicine Pharmacology, Tianjin University of Traditional Chinese Medicine, Tianjin 300193, China; ^3^Zhongnuo R&D Department, CSPC Zhongnuo Pharmaceutical (Shijiazhuang) Co., Ltd., Hebei 050051, China

## Abstract

Xueshuantong for Injection (Lyophilized) (XST), a Chinese Materia Medica standardized product extracted from* Panax notoginseng *(Burk.), is used extensively for the treatment of cerebrovascular diseases such as acutely cerebral infarction clinically in China. In the present study, we evaluated the acute and extended protective effects of XST in different rat cerebral ischemic model and explored its effect on peroxiredoxin (Prx) 6-toll-like receptor (TLR) 4 signaling pathway. We found that XST treatment for 3 days could significantly inhibit transient middle cerebral artery occlusion (MCAO) induced infarct volume and swelling percent and regulate the mRNA expression of interleukin-1*β* (IL-1*β*), IL-17, IL-23p19, tumor necrosis factor-*α* (TNF*α*), and inducible nitric oxide synthase (iNOS) in brain. Further study demonstrated that treatment with XST suppressed the protein expression of peroxiredoxin (Prx) 6-toll-like receptor (TLR) 4 and phosphorylation level of p38 and upregulated the phosphorylation level of STAT3. In permanent MCAO rats, XST could reduce the infarct volume and swelling percent. Moreover, our results revealed that XST treatment could increase the rats' weight and improve a batch of functional outcomes. In conclusion, the present data suggested that XST could protect against ischemia injury in transient and permanent MCAO rats, which might be related to Prx6-TLR4 pathway.

## 1. Introduction

Stroke is harmful to human health and has a high incidence. It is a leading cause of death and the third cause of disability. The loss of neurological functions following stroke is caused by massive loss of neurons resulting from ischemic insults. Up to now, tissue plasminogen activators (tPAs) are still the only agents approved by the Food and Drug Administration since 1996 [1]. However, tPA is currently used in fewer than 5% of stroke victims [2], because of its narrow therapeutic window and risks of cerebral hemorrhage [3]. Therefore, the development of a new drug for stroke with increasing efficacy remains an urgent priority.

More and more evidence suggests that immunomodulation plays an irreplaceable role in postischemic neuroinflammation, which could be a consequence of toll-like receptor (TLR) activation [4]. And the blockade of TLR4 is intimately associated with experimental and clinical outcome in ischemic stroke [4, 5]. Peroxiredoxin (Prx) 6 is newly identified TLR4-dependent inducers of infiltrating macrophage activation and the subsequent production of inflammatory mediators from invading T cells in the ischemic brain [6, 7]. Increased Prx6 was reported to be the major danger associated molecular patterns (DAMPs) that induce infiltrating macrophage activation and the subsequent production of cytokines from invading T cells after cerebral ischemia [8] through TLR4 signal pathway. Therefore, Prx6-TLR4 signaling pathway is believed to be an oval therapeutic strategy for poststroke neuroinflammation and brain injury [4, 9].

In contrast with Western developed countries, China has many herbs and herbal standardized preparations for the clinical treatment of ischemic stroke. San-chi, the root of* Panax notoginseng *(Burk.), is one of the most famous traditional Chinese medicinal herbs. It is widely used to cure heart disease [10], ischemic stroke [11], and acute intracerebral hemorrhage [12] and relieve pain [13] in China or other oriental countries for thousands of years.* Panax notoginseng* saponins are believed to be main active components of San-chi [14, 15]. Xueshuantong for Injection (Lyophilized) (XST) is a standardized product extracted from San-chi. It is recorded by the People's Republic of China Pharmacopoeia and is used extensively for the treatment of cardiovascular disease, cerebrovascular disease, and diabetes in China, with total sales over $700 million in 2013 [16]. Its ingredients, which are several kinds of* Panax notoginseng* saponins, are definite and clear (see Section 2.1). For being accepted worldwide, it demands more strict and accurate evidence for its effectiveness and the principles behind it. In the present study, we evaluated the acute and extended protective effects of XST in different rat cerebral ischemic model and explored its effect on Prx6-TLR4 signaling pathway.

## 2. Materials and Methods

### 2.1. Drug

XST was got from Wuzhou Pharmaceutical Co., Ltd. (Guangxi province, China). The manufacturing technology was taken according to “Pharmacopoeia of China 2010.” The rhizome comminution of* Panax notoginseng *(Burk.) was extracted by 60% ethanol under reflux for 3 times (3 hours per time). The extracting solution was merged for decompressing concentration till there was no alcohol taste and it was further subjected to a D101 macroporous absorption resin column eluted with water, 80% ethanol. The 80% ethanol extract was dried under vacuum to obtain XST. Previous studies have fully studied the main compositions and contents of this preparation [17, 18]. HPLC fingerprint shows that it contains ginsenosides Rg1 48.1%, ginsenosides Rb1 27.8%, notoginsenoside R1 11.1%, ginsenosides Re 5.5%, ginsenosides Rd 1.3%, notoginsenoside Ra 1.1%, and 20-O-glucoginsenoside Rf 0.7% [17, 18]. In this study, XST was freshly prepared in 0.9% normal saline before use.

### 2.2. Animals

Male healthy Wistar rats (260 ± 10 g) were purchased from Vital River Laboratory Animal Technology Co. Ltd. (Beijing, China). The animals were housed under light-controlled conditions, 12 h light/12 h dark cycle, and at room temperature of 22°C. Food and water were available freely. All animal procedures were performed according to the Animal Care and Use Committee at Tianjin University of Traditional Chinese Medicine in China.

### 2.3. Middle Cerebral Artery Occlusion

Anesthesia was induced with chloral hydrate (10%, 3 mL/kg). All groups except sham group underwent focal ischemia surgery induced by left middle cerebral artery occlusion (MCAO) in rats using the intraluminal filament technique as described previously [19]. Briefly, the left common carotid artery was exposed and a silicon-coated 4-0 nylon filament was introduced into the left internal carotid artery through the external carotid artery. The intraluminal suture was carefully withdrawn to establish reperfusion after 90 minutes of ischemia to establish transient MCAO model. For permanent MCAO model, the intraluminal suture was not withdrawn. The sham group received similar surgical procedures without occlusion of the middle cerebral artery. Laser Doppler (Perimed, Jarfalla, Sweden) was used to monitor cerebral blood flow (CBF) during the procedure to confirm occlusion and excluded the rats with a decline rate less than 70%. Body temperature was monitored by a rectal probe and maintained at 37°C using a feedback-regulated heating system (CMA/Micro Dialysis AB, Knivsta, Sweden) during surgery.

### 2.4. Drug Administration

The rats were randomly divided into the following 6 groups: sham group, model group, Xueshuantong 25 mg/kg group (XST-25), Xueshuantong 50 mg/kg group (XST-50), Xueshuantong 100 mg/kg group (XST-100), and edaravone group (as a positive control drug, 6 mL/kg). 50 mg/kg dose was converted from a commonly used dosage of XST in clinical practice in our study.

*Experiment I*. To examine the acute neuroprotection of Xueshuantong on transient MCAO and the mechanism of this effect, Xueshuantong was administrated i.v. immediately after ischemia in transient MCAO rats once a day continuously for 1 or 3 days with 6 groups: sham group (*n* = 9, none died), model group (*n* = 20, 3 died), XST-25 (*n* = 10, 2 died), XST-50 (*n* = 10, 2 died), XST-100 (*n* = 21, 4 died), and edaravone group (*n* = 9, 1 died). There was no statistical difference on mortality between these groups. Experiment I assessed infarct volume, neurological score, hemispheric swelling, mRNA expressions of cytokines, and signaling pathway at 24 h or 72 h after tMCAO.

*Experiment II*. To test the effect of the medicine in permanent cerebral ischemia model, which is more clinically meaningful than transient cerebral ischemia model [20], Xueshuantong was administrated i.v. 4 hours after ischemia in permanent MCAO rats with 4 groups: model group (*n* = 11), XST-25 (*n* = 11), XST-50 (*n* = 12), and XST-100 (*n* = 11). Eight rats survived in all groups. There was no statistical difference in mortality between these groups. Experiment II measured infarct volume and hemispheric swelling at 24 h after pMCAO.

*Experiment III*. To assess the effect of Xueshuantong on long-term functional outcomes, Xueshuantong was administrated i.v. 4 hours after ischemia in transient MCAO rats once a day continuously for 14 days with 2 groups: model group (*n* = 12) and XST-100 (*n* = 12). The survival rates at 1, 3, 7, 14, and 28 d after tMCAO were shown in Section 3.5. Experiment III identified a batch of functional outcomes at 1–28 d after surgery.

### 2.5. Neurological Deficits

The neurological score was used to assess general neurological status [21]. Tests were performed at 24 and 72 hours after surgery in Experiment I and days 1, 3, 7, 14, and 28 after surgeries in Experiment III. The test was scored as follows: 0, no observable deficit; 1, forelimb flexion; 2, decreased resistance to lateral push (and forelimb flexion) without circling; 3, same behavior as 2 but with circling.

### 2.6. Determination of Infarct Volume and Swelling Percent by TTC Staining

After 72 hours of ischemia in Experiment I and 24 h in Experiment II, the rats were deeply anesthetized and brains were rapidly removed, frozen at −20°C for 15 minutes. Seven sections (2 mm thick) were cut using a rodent brain matrix and were stained with 2% (w/v) 2,3,5-triphenyltetrazolium chloride (TTC; Sigma, St. Louis, MO, USA). Infarct volume was analyzed with ImageJ software (Wayne Rasband, National Institutes of Health, USA). Swelling percent was calculated with the formula: swelling percent (%) = (ipsilateral ischemic hemisphere volume − contralateral ischemic hemisphere volume)/contralateral ischemic hemisphere volume × 100%.

### 2.7. Quantitative Real-Time Polymerase Chain Reaction

72 hours after MCAO, rats of sham, model, and XST-100 group (*n* = 6, resp.) in Experiment I were deeply anesthetized and perfused through the heart with cold PBS. All infarct area and penumbra were taken and mixed. Total RNA was isolated using Trizol reagent and processed for cDNA, followed by quantitative real-time polymerase chain reaction (PCR) as described previously [22] The specific primer pairs (Sangon Technology Co. Ltd., Shanghai, China) are listed in Table 1. The mRNA levels of inflammatory mediators were normalized to the value of GAPDH, and the results were expressed as fold change of the threshold cycle (Ct) value relative to sham-operated controls using the 2^−ΔΔCt^ method.

### 2.8. Western Blot Analysis

Twenty-four hours after MCAO, rats of sham, model, and XST-100 group (*n* = 3, resp.) in Experiment I were deeply anesthetized and perfused through the heart with cold PBS. Then protein was extracted and concentration was determined. Samples were electrophoresed in SDS/PAGE gels and transferred onto a PVDF membrane and incubated overnight at 4°C with appropriate primary antibody of p38, phospho-p38, ERK, phospho-ERK, JNK, phospho-JNK, STAT3, phospho-STAT3, Prx6, TLR4, and *β*-actin (Cell Signaling, USA). After incubation with horseradish peroxides' conjugated secondary antibodies (Zhongshan-Golden Bridge, China) for 1 h at room temperature, the blots were developed with chemiluminescence reagent using an ECL kit (Millipore, USA).

### 2.9. Functional Assessment

All rats in Experiment III received EBST on days 1, 3, 7, 14, and 28 after surgery, and Forelimb Placing test on days 3, 7, 14, and 28 after surgery, and Foot Fault, Rotarod, and Y-maze test on day 28 after surgery in a blinded manner. Two hours before surgery, rats were assessed to obtain preinjury baselines.

*Elevated Body Swing Test (EBST)*. The swing test is a simple and easy behavioral test that only requires handling the animal by its tail and recording the direction of swings made by the animal for a certain period of time as described previously [23]. A swing was recorded whenever the animal moved its head out of the vertical axis more than 10° to either side.

*Forelimb Placing Test*. Independent testing of each forelimb for placing in response to visual, vibrissae, tactile, and proprioceptive stimulation is conducted using a test designed by De Ryck et al. [24]. The number of completed placing responses out of 3 for each test was recorded. Average placing scores of testing were calculated for each animal.

*Foot Fault Test.* The Foot Fault test has been shown to be a sensitive indicator for detecting impairments of sensorimotor function after ischemia in rodents and requires very little pretraining. Briefly, an animal is placed on an elevated, leveled grid with openings. Each time a paw slips through an open grid, a “foot fault” is recorded [25]. The number of ipsilateral faults for each limb was recorded.

*Rotarod*. This Rotarod test was to detect motor ability. The rat was practiced for 60 seconds at 4 rpm and test sessions consisted of 3 trials at 10 rpm [26]. If the rats managed to maintain their balance for 300 seconds, the trial was ended. The final score was expressed as the mean time that a rat was able to remain on the rod for the 3 trials.

*Y-Maze*. Y-maze is used as a measure of working spatial memory to assess spontaneous alternation. Briefly, each rat was placed at the end of one arm and allowed to move through the maze for 8 minutes. The percentage of alternation was counted [27].

### 2.10. Statistics

For statistical analysis, a standard software package (SPSS for Windows 19) was used. All data were given as mean ± standard error (SE). For survival data, which is count data, Fisher's exact test was used. Except for survival rate, all other data were examined for assumption of normality using the Kolmogorov-Smirnov test and for homogeneity of variance using Levene's test. For the data with normality and homogeneity of variance, independent-samples *t*-tests (two-group comparison) or one-way ANOVA with Dunnett's posttests (multigroup comparisons) were used. For nonnormal or unequal variances data, Mann-Whitney *U* tests were performed. *P* values < 0.05 were considered significant.

## 3. Results

### 3.1. XST Treatment Decreased Cerebral Infarct Size and Swelling Percent and Improved Neurological Deficits in Transient MCAO Rats

**Figure 1:**
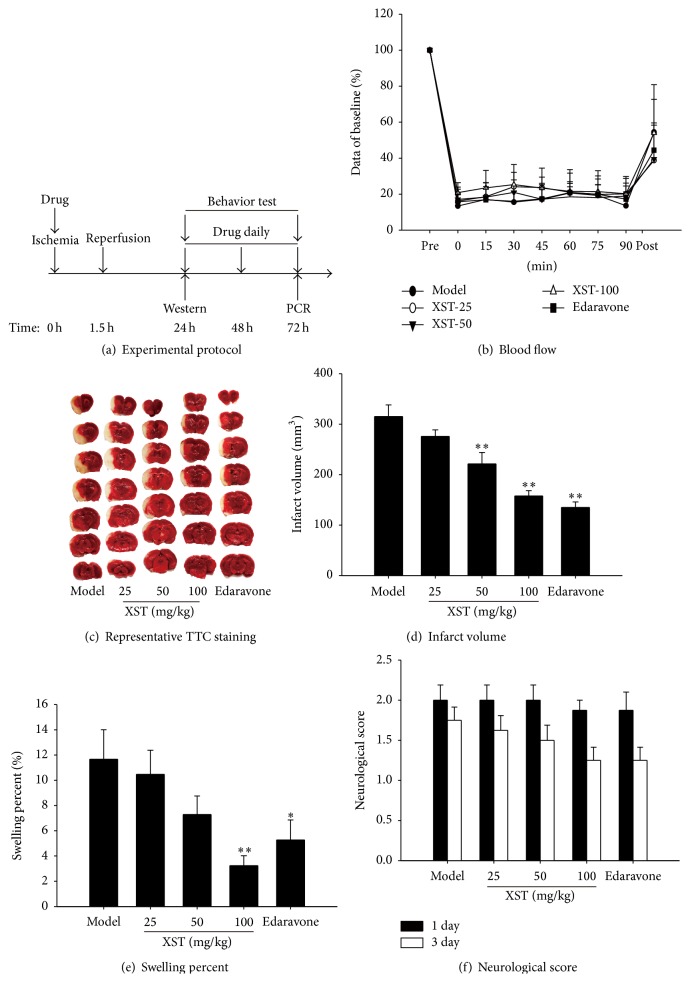
Administration with XST immediately after ischemia attenuated cerebral infarct volume and swelling percent and improved behavioral score in transient MCAO rats. (a) The experiment I protocol. (b) Blood flow of rat brain during the surgery. (c) Representative photographs of TTC-stained coronal brain sections that show viable (red) and dead (white) tissue 72 h after surgery. (d) Cerebral infarct volume expressed as whole infarct area. (e) Swelling percent expressed as a percentage of contralateral brain. (f) Neurological score of rat on days 1 and 3 after surgery. *n* = 8. ^*∗*^ *P* < 0.05, ^*∗∗*^ *P* < 0.01, compared with model group.

Firstly, we evaluated the effects of XST i.v. immediately after ischemia in transient MCAO rats by cerebral infarct volume, swelling percent, and neurological deficits (Figure 1(a)). Laser Doppler flowmetry was used to measure cerebral blood flow during ischemia and reperfusion in transient MCAO rats (Figure 1(b)). Representative photographs of TTC-stained coronal brain sections that show viable (red) and dead (white) tissue 72 h after MCAO were shown in Figure 1(c). Compared with the vehicle-treated model group, XST-50, XST-100, and edaravone group showed notably smaller infarct volumes (Figure 1(d)) and swelling percent (Figure 1(e)). The neurobehavioral tests revealed that XST-100 and edaravone group could also reduce the neurological scores compared with the model group, but without statistical difference (Figure 1(f)), in day 3 after surgery. Edaravone was regarded as positive control drug which has been shown to have neuroprotective effects in animal MCAO experiments [28].

### 3.2. XST Treatment Inhibited mRNA Expressions of Inflammatory Cytokines in Transient MCAO Rats

**Figure 2:**
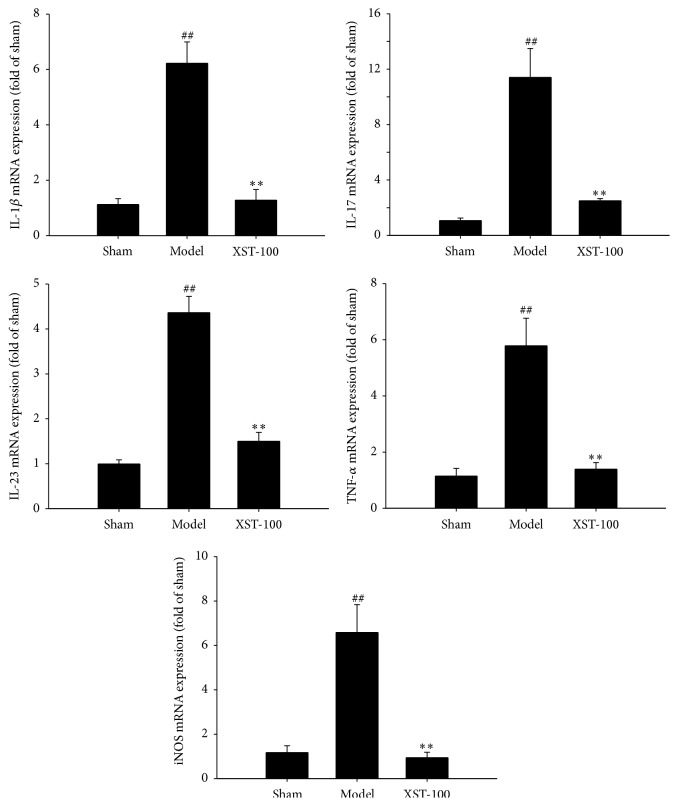
Administration with XST immediately after ischemia (the experiment I protocol) decreases the mRNA expressions of IL-1*β*, IL-17, IL-23p19, TNF*α*, and iNOS in ipsilateral ischemic hemisphere. *n* = 6. ^##^ *P* < 0.01, compared with control group; ^*∗∗*^ *P* < 0.01, compared with model group.

We further observed the changes in mRNA levels of interleukin-1*β* (IL-1*β*), IL-17, IL-23p19, tumor necrosis factor-*α* (TNF*α*), and inducible nitric oxide synthase (iNOS) in brain by real-time PCR analysis. Our results showed that ischemia attack significantly upregulated the mRNA levels of IL-1*β*, IL-17, IL-23p19, TNF*α*, and iNOS, respectively. XST treatment reversed the increase of mRNA levels of these cytokines (Figure 2).

### 3.3. XST Treatment Inhibited the Expression of Prx6-TLR4 Signaling Pathway and Upregulated the Phosphorylation Level of STAT3 in Transient MCAO Rats

**Figure 3:**
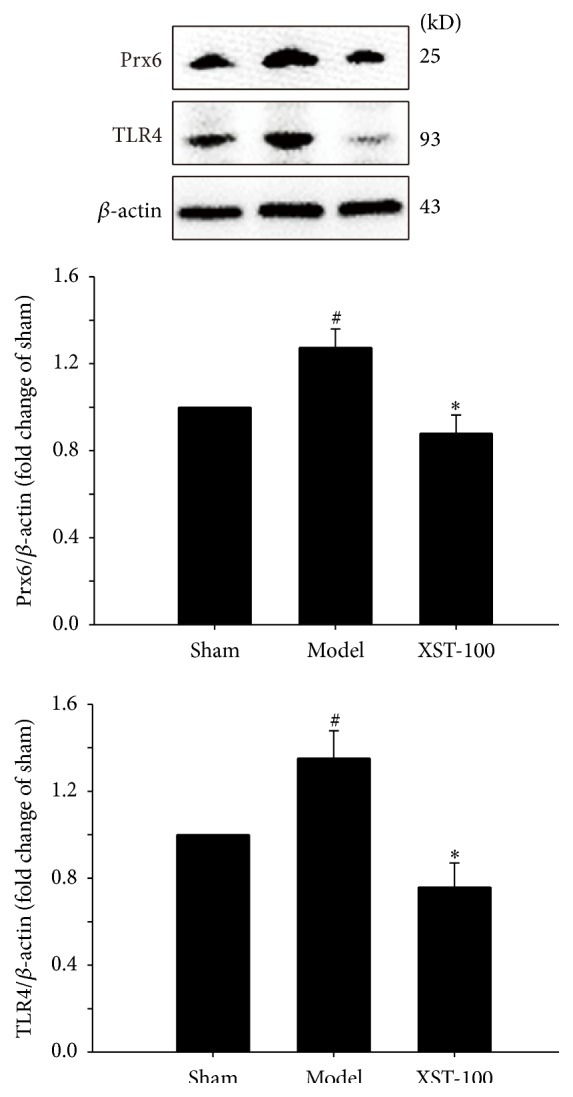
Administration with XST immediately after ischemia (the experiment I protocol) reduced the expression of TLR4 and Prx6 in ipsilateral ischemic hemisphere. Representative Western blots and quantitative analysis of Prx6 and TLR4 expression in the ischemic brain tissues 24 h after transient MCAO. The ratio of Prx6 and TLR4 to *β*-actin was densitometrically analyzed and expressed as the relative optical density of the sham group. *n* = 3. ^#^ *P* < 0.05, compared with control group; ^*∗*^ *P* < 0.05, compared with model group.

**Figure 4:**
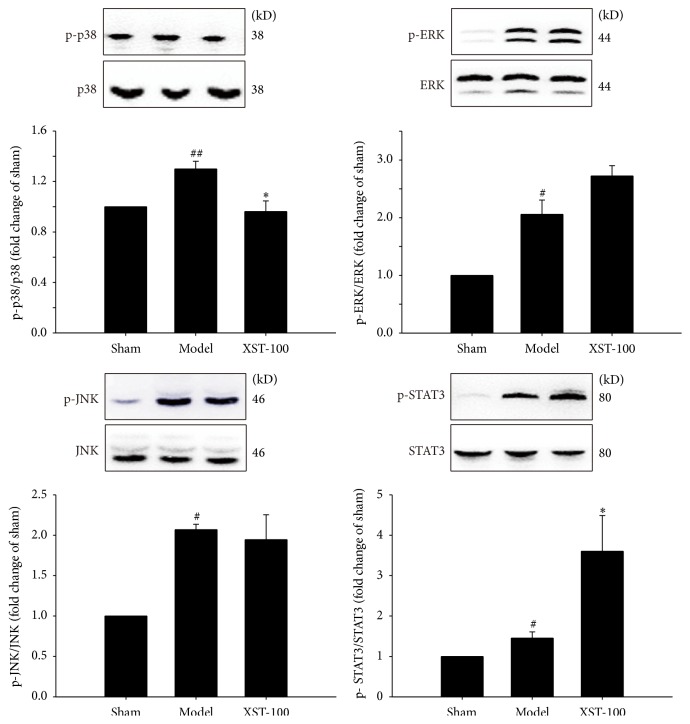
Administration with XST immediately after ischemia (the experiment I protocol) reduced the phosphorylation level of p38 and increased the phosphorylation level of STAT3 in transient MCAO rat brain. Representative Western blots and quantitative analysis of phosphorylation levels of p38, ERK, JNK, and STAT3 in the ischemic brain tissues 24 h after MCAO. The phosphorylation levels of p38, ERK, JNK, and STAT3 were densitometrically analyzed and expressed as the relative optical density of the sham group. *n* = 3. ^#^ *P* < 0.05, ^##^ *P* < 0.01, compared with control group; ^*∗*^ *P* < 0.05, compared with model group.

As shown in Figure 3, the expression levels of Prx6 and TLR4 in transient MCAO rats were markedly increased in the model group compared with those in the sham groups, while XST treatment could decrease the expression levels of Prx6 and TLR4 compared with model groups 24 h after MCAO. To elucidate the mechanism of the neuroprotective effects of XST against ischemia brain injury, we further explored the effect of XST on the MAPK signaling pathway in transient MCAO rat brain. As shown in Figure 4, cerebral ischemia significantly induced the subsequent activation of TLR4 signaling effectors, reflected by increasing the phosphorylation levels of p38, ERK, and JNK in the ischemic brain in model group compared with control groups. XST-100 inhibited the phosphorylation level of p38. Phosphorylation of STAT3 is reported to reduce the inflammatory injury through inhibition of Ras-MAPK pathway activation [29]. As shown in Figure 4, the phosphorylation level of STAT3 in the ischemic brain in model group was upregulated. XST could increase the phosphorylation level of STAT3 compared with model groups.

### 3.4. XST Treatment Decreased Cerebral Infarct Size and Swelling Percent in Permanent MCAO Rats

**Figure 5:**
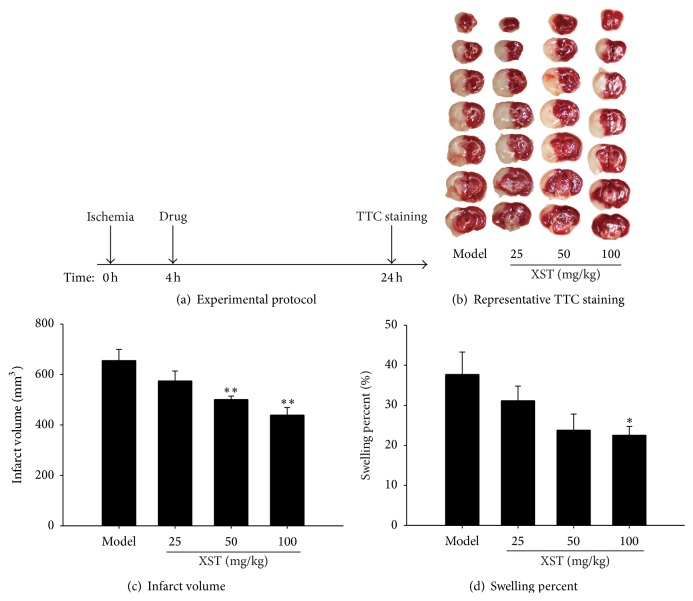
Administration with XST 4 hours after ischemia attenuated cerebral infarct volume and swelling percent in permanent MCAO rats. (a) The experiment II protocol. (b) Representative photographs of TTC-stained coronal brain sections 24 h after MCAO. (c) Cerebral infarct volume expressed as whole infarct area. (d) Swelling percent expressed as a percentage of contralateral part. *n* = 8. ^*∗*^ *P* < 0.05, ^*∗∗*^ *P* < 0.01, compared with model group.

Next, we evaluated the brain infarct volume and swelling percent in permanent MCAO rats. XST was administrated i.v. at 4 h after ischemia in permanent MCAO rats, which were sacrificed at 24 h after ischemia (Figure 5(a)). Representative photographs of TTC-stained coronal brain 24 h after MCAO were shown in Figure 5(b). Treatment of XST significantly decreased the infarct volume (Figure 5(c)) and swelling percent (Figure 5(d)) in a dose-dependent manner.

### 3.5. XST Treatment Improved Long-Term Functional Outcome in Transient MCAO Rats

**Figure 6:**
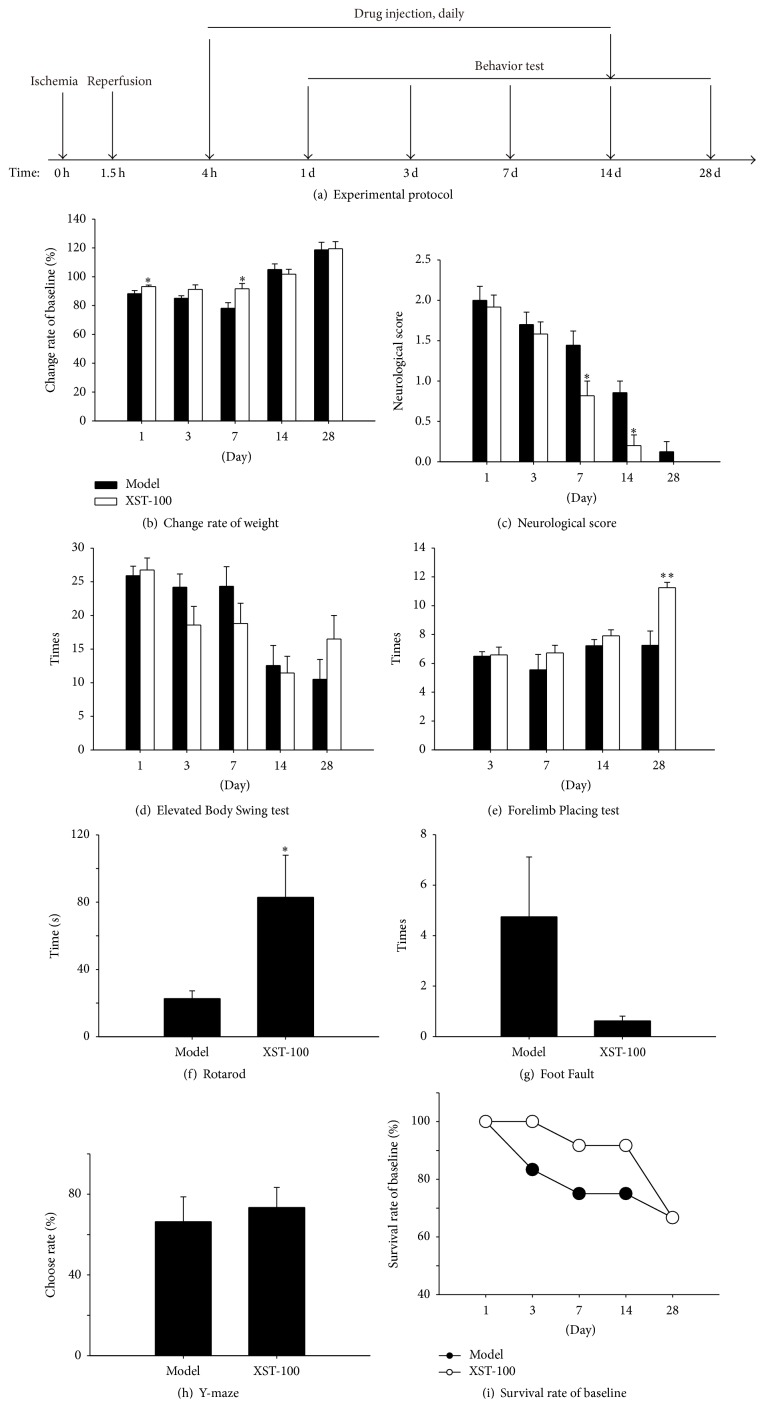
XST treatment improves long-term functional outcome in transient MCAO rats. Administration with XST 4 hours after ischemia improved long-term functional outcome after 90 min ischemia in transient MCAO rats. (a) The experiment III protocol. (b) Change of weight from days 1 to 28 after surgery. (c) Neurological score from days 1 to 28 after surgery. (d) Elevated Body Swing test (EBST) from days 1 to 28 after surgery. (e) Forelimb Placing test from days 3 to 28 after surgery. (f) Rotarod test on day 28 after surgery. (g) Foot Fault test on day 28 after surgery. (h) Y-maze test on day 28 after surgery. (i) Survival rate of baseline from days 1 to 28 after surgery. *n* = 8–12. ^*∗*^ *P* < 0.05, ^*∗∗*^ *P* < 0.01, compared with model group.

Six functional assessments, including neurological score, Forelimb Placing test, EBST, Rotarod test, Foot Fault test, and Y-maze test, were performed at days 1, 3, 7, 14, and 28 after surgeries to evaluate long-term functional outcome of the treatment of XST (Figure 6(a)). XST-100 could significantly increase body weight after being administrated at 1 and 7 days after MCAO compared with model group (Figure 6(b)). XST-100 could decrease neurological score on days 7 and 14 after surgery (Figure 6(c)), improve Forelimb Placing test score on days 3 and 28 (Figure 6(e)), and lengthen movement time on day 28 (Figure 6(f)). Compared with model group, XST-100 group showed no significant change in EBST (Figure 6(d)), Foot Fault test (Figure 6(g)), Y-maze test (Figure 6(h)), and survival rate (Figure 6(i)).

## 4. Discussion

Stroke is a serious disease for human being. There is a great demand for intervention therapy. Unfortunately, although more than 700 drugs that target cerebral ischemia showed beneficial effects in preclinical animal studies, none of them proved efficacious in treating stroke patients in the past several decades [30]. This implies to us that one-target treatment for stroke was impossible [31, 32]. Meanwhile XST is a natural mixture, extracted from San-chi, composed of several components, such as ginsenosides Rb1, Rg1, Rd, Re, and* P. notoginseng* saponin R1. It is reported that these components have showed neuroprotection through a variety of mechanisms. Rb1 can weaken the activity of microglia, decrease the upregulation of brain tissue mRNA of TNF*α* and interleukin-1 (IL-1), IL-*β*, and IL-6, and decrease expression of cox-2 mRNA and protein content in the brain induced by systemic lipopolysaccharide (LPS) treatment in C57BL/6 mice [33]. Rg1 can decrease the expression of c-fos gene and protein in the hippocampus of aged rats [34] and improve the learning and memory function of rats with electrical hippocampal injury [35]. Ginsenoside Rd can attenuate redox imbalance and improve stroke outcome after focal cerebral ischemia in aged mice and attenuate mitochondrial dysfunction and sequential apoptosis after transient focal ischemia by reducing inflammatory response and protecting mitochondria [36, 37]. Ginsenoside Re can reduce the MDA content and the apoptosis of H^+^-ATP to protect the neurons from ischemia injury in MCAO rats [38].* P. notoginseng* saponin R1 can protect viability of rat neural stem cells and neuronal cells from glutamate interference in primary cultured mouse cortical neurons [39]. Based on these bioactive compounds, we suppose that XST could prevent against ischemic stroke through various ways, although our study just explored one signaling pathway.

Most of the time, many studies choose infarct volume to evaluate the neuroprotective effect of a drug, ignoring the long-term assessment, which leads to a failure in translation to clinical practice. In the present study, we explored the neuroprotective effect of XST in the acute and long-term functional outcome after ischemia. Our data showed that treatment with XST immediately after ischemia resulted in a significantly smaller infarct volume and less swelling percent than vehicle-treated model rats in transient MCAO. Importantly, treatment with XST 4 hours after onset of ischemia reduced infarct size and swelling percent in permanent MCAO rats. What is more, functional recovery is the final aim of treatment and we chose a batch of functional assessments to evaluate functional recovery. We found that administration of XST continuously for 14 days improved long-term functional outcome compared with model group. Our results also showed that although 50 mg/kg dose of XST was converted from a commonly used dosage of XST in clinical practice, XST-100 was the most effective volume in our animal experiment. It suggested that the dose can be raised for a better prognosis. By the way, to confirm our MCAO model and experimental system, we used edaravone as a positive control, which has been proved to be neuroprotective in several rodent MCAO models by many researchers [28, 40, 41]. Our results were consistent with these previous reports [28, 40, 41].

More and more evidence suggests that both innate and adaptive immunity, highly regulated by TLR/danger associated molecular patterns (DAMPs) signaling, play an irreplaceable role in the progression of postischemic neuroinflammation and brain injury. The binding of DAMPs to TLRs induces innate immune responses through the activation of TLR signaling effectors, including mitogen activated protein kinase (MAPK), resulting in the production of proinflammatory mediators, such as IL-1*β*, IL-17, IL-23p19, TNF*α*, and iNOS [42, 43]. Prx's were originally described as antioxidative enzymes within brain cells that exert neuroprotective effects by scavenging reactive oxygen species [44]. However, once released from postischemic dead or dying neural cells, Prx's lose antioxidant capacity, act as DAMPs of TLRs, and induce innate and adaptive immune responses by activating TLR signaling, triggering neuroinflammation and brain lesions in ischemic stroke [45]. A recent study identified extracellular Prx5 and Prx6 as major TLR2/TLR4-dependent DAMPs in aseptic inflammation after cerebral ischemia [46]. Therefore, immunomodulation through TLR/DAMP signaling is a potential therapeutic approach for neuroinflammation and brain injury after ischemic stroke. In this study, we observed the activation of cerebral ischemia induced TLR4 signaling, including enhancing the protein expression of Prx6 and TLR4, and the activation of MAPK signal pathway. Administration os XST resulted in less mRNA expression of inflammatory cytokines IL-1*β*, IL-17, IL-23p19, TNF*α*, and iNOS, as well as preventing the upregulation of Prx6, TLR4, and the phosphorylation levels of p38 in transient MCAO rats.

The phosphorylation of STAT3 was reported to suppress the activation of MAPK pathway [29]. Administration of XST could highly increase the phosphorylation levels of STAT3. We suppose that XST could also inhibit MAPK pathway by the phosphorylation of STAT3.

We did not explore intracerebral distribution of XST in this paper. Previous study showed that the original type of panaxadiol saponin of* Panax* ginseng and protopanaxatriol can be detected in brain after i.v. (30 mg/kg) administration in vivo, and their concentrations in hippocampal were relatively higher than that in cortex [47]. My team group found that the components of XST were easier to pass blood brain barrier after cerebral ischemia. For example, after administration of XST (i.v., 100 mg/kg) 24 h after surgery, the concentration of ginsenoside Rg1 in ischemic cortex was 15 times in MCAO rats more than in sham group (unpublished).

## 5. Conclusions

In conclusion, our data showed that (1) treatment with XST (50, 100 mg/kg) immediately after ischemia resulted in significant neuroprotection against acute brain injury in a transient MCAO rat model dose-dependently; (2) treatment with XST (50, 100 mg/kg) 4 hours after onset of ischemia reduced infarct size and swelling percent in permanent MCAO rats in a dose-dependent manner; (3) administration of XST (100 mg/kg) continuously for 14 days improved long-term functional outcome compared with model group. Moreover, the neuroprotective mechanism of XST might be related to prevention of expressions of inflammatory cytokines and Prx6-TLR4 pathway.

## Figures and Tables

**Table 1:** The specific primer pairs used in polymerase chain reaction.

Gene	Accession number	Primer pair (5′-3′); F: forward; R: reverse
GAPDH	NM017008.4	F: CCCCCAATGTATCCGTTGTG
		R: TAGCCCAGGATGCCCTTTAGT

IL-1*β*	M98820.1	F: GAAGTCAAGACCAAAGTGG
		R: TGAAGTCAACTATGTCCCG

IL-23p19	NM130410.2	F: AAAGGAGGTTGATAGAGGGT
		R: TCTTAGTAGATCCATTTGTCCC

IL-17	NM001106897.1	F: CACAAGCTCATCCCGTACCA
		R: CAGGCACATGGATGGAATTCT

TNF*α*	HQ201305.1	F: TCTTCTCATTCCTGCTCGTGG
		R: GGTCTGGGCCATGGAACTGA

iNOS	XM003750865.2	F: AAAATGGTTTCCCCCAGTTC
		R: GTCGATGGAGTCACATGCAG
